# Influence of High-Pressure Torsion Processing on the Tribological Properties of Aluminum Metal Matrix Composites

**DOI:** 10.3390/ma16010216

**Published:** 2022-12-26

**Authors:** Mohamed Ibrahim Abd El Aal, Hossam Hemdan El-Fahhar, Abdelkarim Yousif Mohamed, Elshafey Ahmed Gadallah

**Affiliations:** 1 Mechanical Engineering Department, College of Engineering at Wadi Addawaser, Prince Sattam Bin Abdulaziz University, Wadi Addawaser 18734, Saudi Arabia; 2 Mechanical Design & Production Department, Faculty of Engineering, Zagazig University, Zagazig 44519, Egypt; 3 Mechanical Production Department, Faculty of Technology & Education, Suez University, Suez 43527, Egypt

**Keywords:** Al metal matrix composites (Al MMCs), high-pressure torsion (HPT), wear and frictional properties

## Abstract

The motivation for the current study was to improve the wear and frictional properties of Al, Al–Al_2_O_3_, and SiC MMCs through HPT processing. The wear test using a tungsten carbide (WC) ball was carried out for different PM and HPT-processed Al and MMC samples. The effect of the sample processing methods on the wear rate, friction, and wear surface morphology was thoroughly investigated. The high hardness after Al grain refinement and reinforcement fragmentation through the HPT processing of the samples increased the wear resistance by 16–81% over that of the PM samples. The average coefficient values and variation ranges were reduced after HPT processing. The Al and Al MMC processing methods affected the wear mechanism and surface morphologies, as proven by the microscopic observations and analyses of the worn surfaces of the samples and WC balls.

## 1. Introduction

The motivation from previous studies was the need for high-performance materials required in different industrial applications. The enhancement of traditional materials, especially metals, was obtained through different methods. Among the different processing methods used in the enhancement of the properties of the metals were the formation of the MMCs [1,2,3,4,5,6,7] and the grain refinement through severe plastic deformation (SPD) [8,9,10,11,12,13,14,15,16,17,18,19].

MMC fabrication methods can be classified based on the starting state of the metal matrix. The MMCs fabrication method starting with the liquid-phase matrix, includes different processes, such as casting with different methods. On the other hand, powder metallurgy (PM) is the most popular process among the primary solid-phase processing methods. The disadvantages of using liquid-phase matrix processing methods, such as insufficient wetting of the ceramic particle and high volume of void content, turn the rudder in the direction of using the primary solid-phase methods [4,5].

Moreover, the PM can produce ultrafine fine (UF) and nanostructure MMCs. The PM of UF and nanopowders and the combination of PM and mechanical alloying such as ball milling can effectively produce UF and nanostructure MMCs [6,7]. Furthermore, the low sintering temperatures used in PM can conserve the UF and nano microstructure relative to the higher temperatures used in other methods, such as rapid solidification and different casting methods. Using PM can also be considerably cheaper than modern methods used in the fabrication of MMCs, such as additive manufacturing.

Recently, the fabrication of MMCs and intermetallic samples using different SPD processes improved their mechanical properties [8,9,10,11,12,13,14,15,16,17,18,19,20,21]. The effectiveness of the SPD processing of the MMCs to overcome the defects of PM was previously proven through the HPT processing of Cu MMCs [18]. The HPT processing of MMCs contributes to refining the matrix grains of MMCs [8,9,10,11,12,13,14,15,16,17,18,19,22,23,24] and fragmentation of the reinforcement particles [8,9,10,16,17,18,19,20,21,22,23,24]. Moreover, HPT processing provided a redistribution of the fragmented reinforcement particles with the homogenized distribution.

HPT processing suffers from upscaling limits and a relatively lower deformation homogeneity since its inherent radius depends on the strain. However, processing by HPT has different advantages over other SPD processes, such as permitting a defined continuous variation in strain whereas most SPD processes offer only a stepwise application of strain. Moreover, HPT processing imposes high shear, so HPT processing can effectively process brittle or high-strength materials such as MMCs, which is often impossible using other SPD processes. Further advantages of the HPT process can be found in detail in previous work [25]. The recent work on the HPT processing and PM of Al and Al MMCs proves the capability of HPT processing in producing samples with superior mechanical properties relative to PM [26].

With its high mechanical properties, Al suffers from relatively low wear resistance and tribological characteristics. Al’s low wear resistance was one of the motivations for forming Al MMCs samples [1,2,3,4,27,28]. Fabricating different Al MMCs enhances the hardness and wear resistance of the Al samples [26,27,28,29,30,31,32,33]. Although the formation of the MMC samples improves the wear properties, the decreasing matrix grain and reinforcement particle sizes were also effective methods of improving the metal’s and MMCs’ wear characteristics, especially using different SPD processes [16,34,35,36,37,38,39,40,41,42]. ECAP and HPT processing improve the wear resistance of different Al and Cu alloys and MMCs [16,34,35,36,37,38,39,40,41,42].

Previous works were interested in improving Al, Al alloys, and Al MMCs’ mechanical properties and wear characteristics by forming MMCs or SPD processing [8,9,13,14,19,23,24,26,27,28,29,30,31,32,37,38,39,42]. Unfortunately, no previous studies cover the influence of the HPT process on the wear scars, wear resistance, and friction properties of Al MMCs. Moreover, there was a shortage in the correlation between the wear rate and friction properties from one side and the worn surface morphology. Therefore, a comprehensive study of the effect of HPT processing on different Al MMC tribological characteristics is still required.

The present work aims to:

1. Study the effect of Al MMC sample processing methods on their tribological characteristics.
2. Study the effect of the processing method on the worn surface morphologies of samples, and wear test balls and wear diapers.

## 2. Materials and Methods

The fabrication steps of the Al, Al-10%, and 20% of Al_2_O_3_ and SiC samples using PM and HPT processes were mentioned in detail in previous work [26]. A detailed flow chart summarizing all of the fabrication that could be observed is shown in previous work [26]. Furthermore, the microstructure observations for the PM and HPT-processed samples were present in the previous work [26]. A complete tracing of the matrix grain refinement and reinforcement particle fragmentation and distribution of the PM and HPT-processed samples was introduced [26]. Moreover, microhardness measurement was thoroughly investigated [26] based on the previous work [43].

A dry wear test was performed for the PM and HPT-processed samples to investigate the effect on the wear rate, wear scars, and frictional properties of the different Al MMCs. Reciprocating ball-on-flat surface wear tests were carried out at RT under different sliding distances and with loads of up to 1260 m and 20 N using a tungsten carbide (WC) ball [37,42]. The wear test was carried out at a speed of 0.07 m/s and humidity of 50 ± 5%. In the current research, wear test parameters were near those previously used for different severely deformed Al alloys and composites [32,33,34,35,36,37,42]. The test was repeated five times to obtain highly reliable results. Dispersive X-ray spectroscopy (EDS) analysis and SEM photomicrograph were performed using SEM (model XL30SFEG, Philips, Tokyo, Japan) for the worn surface morphology, WC balls, and wear diapers. 

## 3. Results and Discussion

### 3.1. Wear Characteristics

#### 3.1.1. Wear Scars

**Figure 1:**
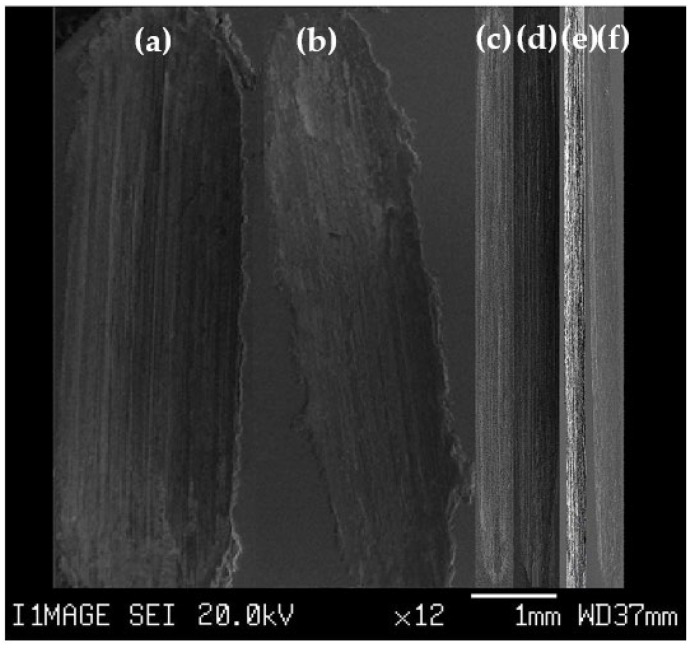
Photomicrographs of the wear scars of (**a**) PM Al, (**b**) HPT-processed Al, (**c**) PM Al-20% SiC, (**d**) PM Al-20% Al_2_O_3_, (**e**) HPT-processed Al-20% SiC, and (**f**) HPT-processed Al-20% Al_2_O_3_ samples tested under a sliding distance of 1260 m and an applied load of 20 N.

The different wear scars obtained under 20 N and 1260 m are shown in Figure 1. The scar width of the PM Al sample decreased from 2075.1 to 1570.1 µm after processing by HPT (Figure 1a,b). The decrease in the wear scar width becomes obvious after the HPT processing of Al MMCs. The width of the wear scar of the HPT-processed Al-20% Al_2_O_3_ and SiC samples decreased to 315.3 and 281.5 µm (Figure 1e,f), with a percent decrease of 80–83% in the width of the wear scars relative to the HPT-processed Al sample. Furthermore, the HPT processing of the Al MMCs decreased the width of the wear scars of the PM MMCs samples by 36–39%. Therefore, the HPT processing of Al MMCs can effectively improve their wear resistance.

The decrease in the width of the wear scars of the Al sample was also noted after forming MMCs and the HPT processing of Al MMCs. The wear scar width of the PM Al sample tested under 5 N and 400 m decreased from 500 µm to 375 and 313 µm with a 25–37% percent decrease after forming Al-5wt.%Ti and Al-5% wt.% A1N Al MMCs [29]. Furthermore, the formation of Al MMCs contributed to a 76–78% decrease in the Al scar width. This decrease was due to the increases in microhardness [26], which reduced the volumetric wear loss due to Archard’s law [44].

As previously noted, the decrease in the scar widths was more obvious after the HPT processing of different Al MMCs [16,39,42]. The wear scar widths of the extruded Al-Si-Cu-5% SiC decreased by 28% after processing by HPT [16]. This observation was also noted after accumulative roll bonding (ARB) of Al-SiC [33] and the HPT processing of Al 99.99%, Al 99.5%, and Al-20% Al_2_O_3_ [39]. The HPT processing of Al-20% Al_2_O_3_ decreases wear scar widths by 45–60% of the HPT-processed Al 99.99% and Al 99.5%. HPT processing of Al chip and Al chip MMCs also decreased the scar widths by 39–76% [42]. The higher microhardness of the HPT-processed Al MMCs [16,39,42] compared to those processed by PM [29,32] leads to a decrease in the wear scar width by 39–76% [16,39,42]. Therefore, increasing microhardness through the HPT processing of Al MMCs [26] can reduce the volumetric wear loss and decrease the wear scar width according to Archard’s law [43,44]. Previous work covers the strengthening mechanisms and increases in hardness of PM and HPT-processed MMCs [18,26].

#### 3.1.2. Wear Rate Results

**Figure 2:**
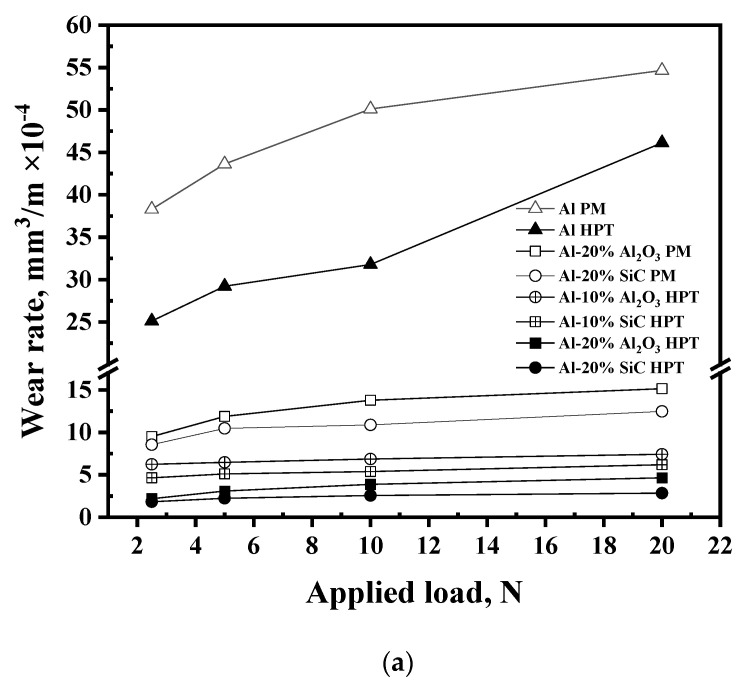

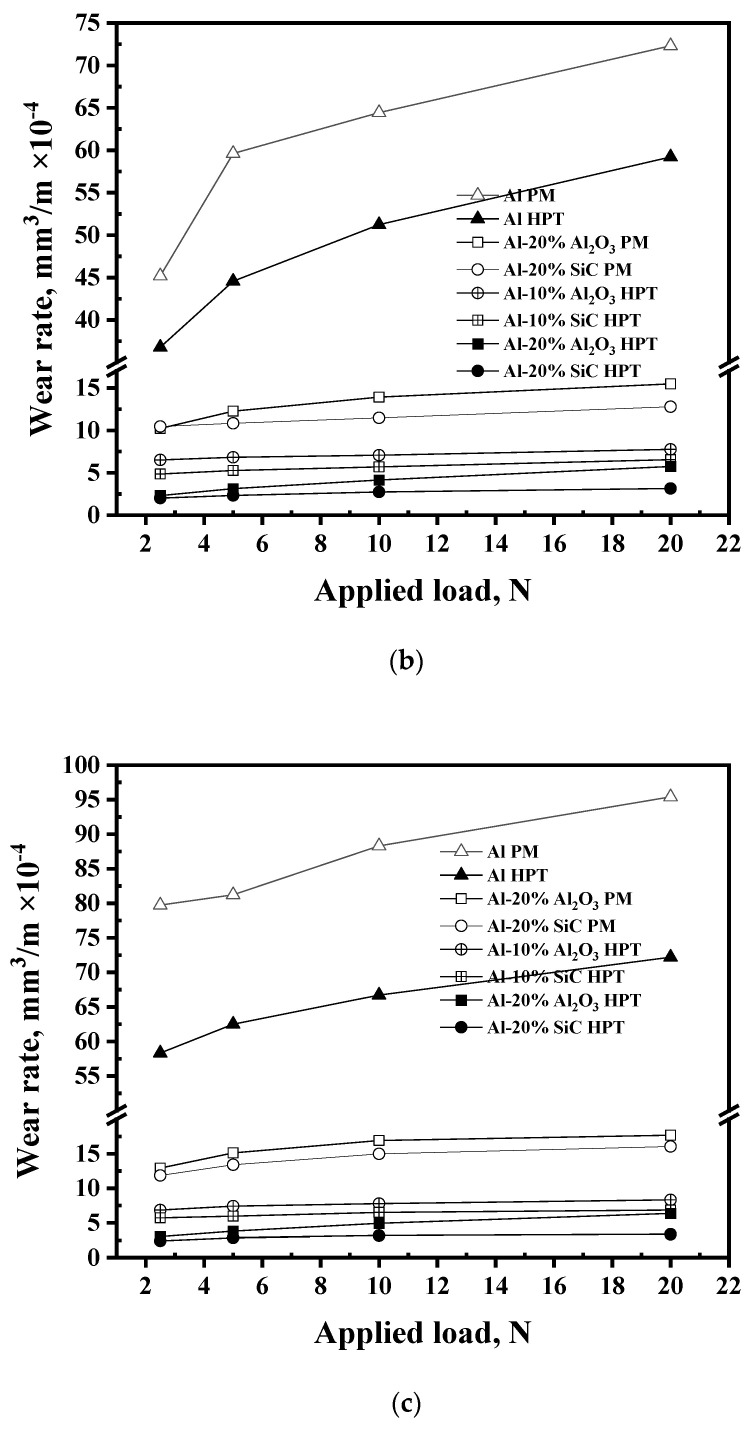
The wear rate curves of the different samples under different applied loads and sliding distances of (**a**) 315, (**b**) 630, and (**c**) 1260 m.

The variation in the wear rate under different distances and loads is shown in Figure 2. The wear rate was increased with increasing distance and load. Increasing the distance from 315 to 630 m and 1260 m increased the wear rate by 1–38% and 9–57% for the different Al and Al MMC samples. On the other hand, the load increase under different sliding distances increased the wear rate by 2–57% for the different Al and Al MMC samples. A similar increase in wear rate with increasing distance and load was also noted for Al and different Al MMCs [42]. The present study is more comprehensive than the previous study in terms of the wear of Al and Al MMCs. The present study covers the wear characteristics through a wide range of loads and distances relative to those previously noted [29,30,31,32,33].

The wear rate was decreased obviously after the formation MMCs and the increase in reinforcement Vol%. The PM and HPT processing of the Al-20% Al_2_O_3_ MMCs decreased the wear rate by 72.2- 83.7 and 87.8–94%, respectively, relative to the PM and HPT Al samples. Moreover, PM and HPT processing of the Al-20% SiC MMCs decreased the wear rate by 76–85.2% and 92–95.8%, respectively, relative to the PM and HPT Al samples. The Al wear rate was decreased by 3.5–62.5% after forming different Al MMCs with different reinforcement volume fractions [29,30,31,32,33]. Increasing the Vol% of both Al_2_O_3_ and SiC also improves the wear resistance. The wear rate of the HPT-processed Al-20% Al_2_O_3_ and SiC MMCs samples decreased by 24–60% and 50–63%, respectively, relative to that of the HPT-processed Al-10% Al_2_O_3_ and SiC MMCs sample. The relationship of increasing reinforcement Vol% with decreasing wear rate was also previously noted [31,32]. 

Interestingly, the HPT processing of Al and Al MMCs effectively improves the wear resistance (Figure 2). The HPT-processed Al and Al MMCs sample wear rates were 16–37, 63–78, and 75–81% lower than those of the PM samples. Furthermore, relative to previous works [28,29,30,31], the HPT-processed Al MMCs samples have a much lower wear rate than the PM and cast Al MMC samples. The wear rates of 2.2 and 3.1 × 10^−4^ mm^3^/min for the HPT-processed Al-20% Al_2_O_3_ and SiC samples under test conditions of 315 m and 5 N applied load were noted to be much smaller than the 2.6 – 3.4 × 10^−4^ mm^3^/min wear rates for Al-5% A1N and Al-5%Ti [29] and the 333.3 mm^3^/min wear rate for the Al-20% Al_2_O_3_ samples in [32] (tested under similar conditions). Moreover, the wear rates for the HPT-processed Al-20% Al_2_O_3_ and SiC samples obtained under 315 m and 10 N applied load were lower than those of Al-1.5% Al_2_O_3_, Al-1.5% Al_2_O_3_ + 4% Al_4C3_, and Al – 1.5% Al_2_O_3_ + 12% Al_4_C_3_ tested under 200 m and 10 N [30]. Furthermore, the wear rate values for the HPT-processed Al-20% Al_2_O_3_ and SiC samples tested under 1260 m and 10 N applied load were 3.2 and 4.9 × 10^−4^ mm^3^/min, respectively, which were also smaller than the 5.5–7.9 × 10^−4^ mm^3^/min wear rate of Al-6% Al_2_O_3_ and Al-6% SiC tested under 1130 m and 10 N applied load conditions [31]. The present observations can be explained by the obvious higher microhardness of the HPT-processed samples [26]. Therefore, the improvement in microhardness is the key factor in improving wear resistance according to Archard’s law [44].

Improving the wear resistance of the Al and Al MMCs through HPT processing in the present work was more effective than those previously noted after the ARB and HPT processing of Al solid, Al powder, and chip MMCs [33,42]. Moreover, the HPT-processed Al-20% Al_2_O_3_ sample in the present work tested under actual conditions reached 1260 m and 20 N applied load, considerably higher than those of 1 m and 10 N used in testing the HPT-processed Al-20% nano Al_2_O_3_ [39]. This is because starting with microsize Al_2_O_3_ reduces the Al_2_O_3_ agglomeration and so the void content that consequently increases the microhardness, as noted in previous work [26], and similarly increases the wear resistance. 

The wear rate of the accumulative roll bonded Al-SiC tested under 100 m, and 50 N applied load conditions [33] was 285 mm^3^/min and was considered much higher than that of 3.4 × 10^−4^ mm^3^/min for the HPT-processed Al-20% SiC sample tested using 1260 m and 20 N applied load. Interestingly, the present work wear rate result for the Al MMCs was comparable to or even lower than that of HPT-processed Al chip MMCs [41]. The present study’s HPT processing under 30 revolutions increases the Al MMC’s microhardness due to smaller grain and particle sizes with lower void content [26]. HPT-processed Al-20% Al_2_O_3_ and SiC samples wear rates were 5–50% lower than HPT-processed Al chip MMCs [42]. Therefore, the HPT processing of Al powder MMC samples can produce samples with superior wear resistance compared to the chip matrix.

### 3.2. Friction Characteristics

**Figure 3:**
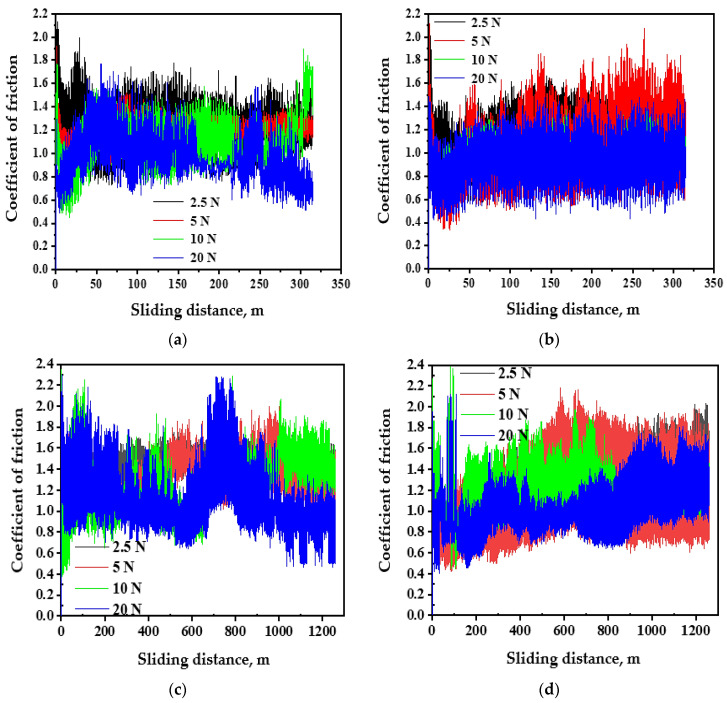
The COF curves of (**a**,**c**) PM Al and (**b**,**d**) HPT-processed Al samples under different applied loads and sliding distances of 315 and 1260 m.

**Figure 4:**
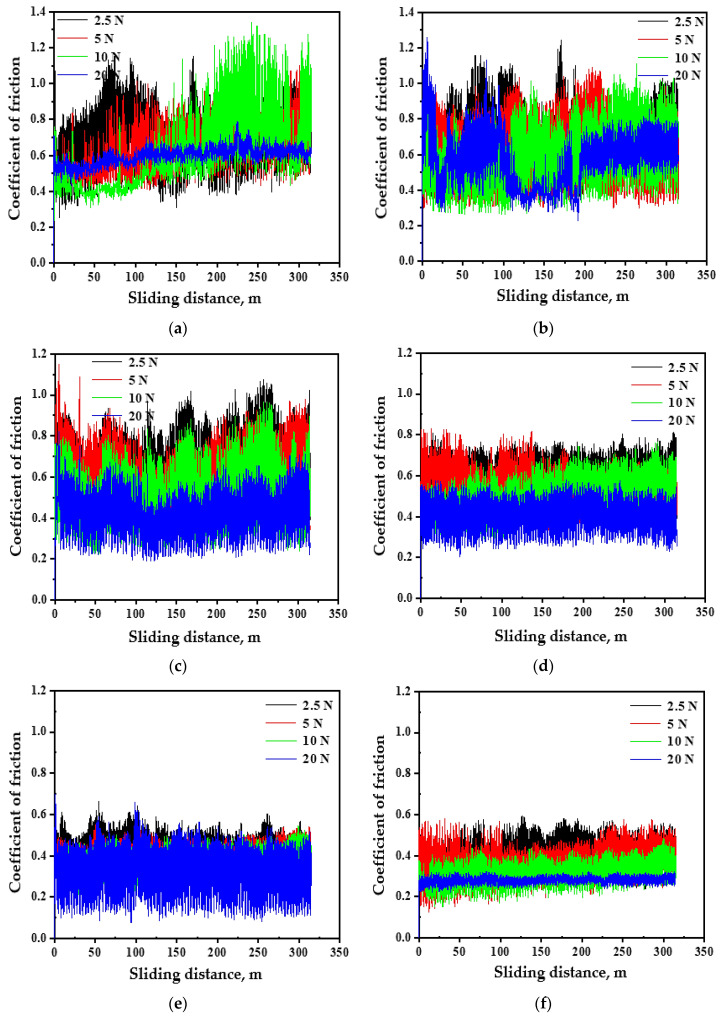
The COF curves of (**a**) PM Al-20% Al_2_O_3_, (**b**) PM Al-20% SiC, (**c**) HPT-processed Al-10% Al_2_O_3_, (**d**) HPT-processed Al-10% SiC, (**e**) HPT-processed Al-20% Al_2_O_3_, and (**f**) HPT-processed Al-20% SiC samples under different applied loads and a sliding distance of 315 m.

**Figure 5:**
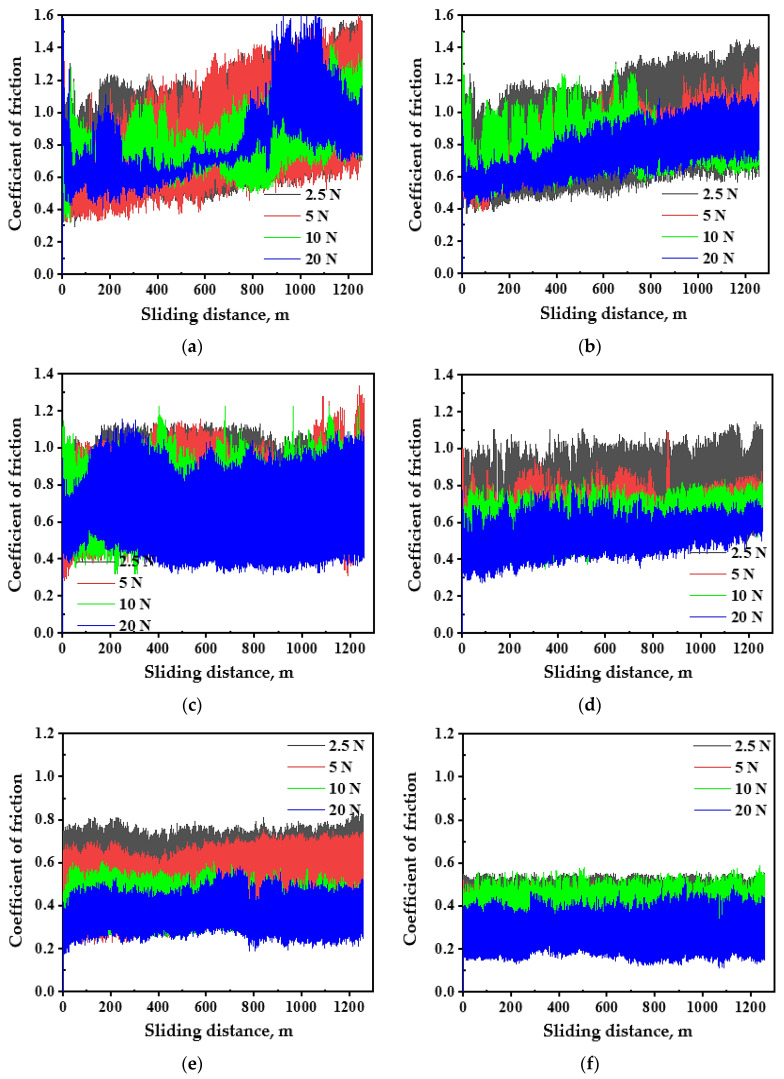
The COF curves of (**a**) PM Al-20% Al_2_O_3_, (**b**) PM Al-20% SiC, (**c**) HPT-processed Al-10% Al_2_O_3_, (**d**) HPT-processed Al-10% SiC, (**e**) HPT-processed Al-20% Al_2_O_3_ and (**f**) HPT-processed Al-20% SiC samples under different applied loads and a sliding distance of 1260 m.

The coefficient of friction (COF) behavior of PM and HPT-processed samples under different distances and loads are shown in (Figure 3, Figure 4 and Figure 5). The COF of the PM and HPT-processed Al has approximately the same behavior under different conditions, as it increased from zero and then oscillated with different amplitudes (Figure 3). HPT-processed Al samples have a slightly narrower span of COF variation under different conditions relative to the PM Al samples. This difference also appears through the smaller average COF values of the HPT-processed Al samples. The HPT-processed Al sample average COF was 4–12% lower than the PM Al average COF due to higher microhardness [26].

The PM and HPT-processed Al MMC COF behaviors during the wear tests (Figure 4 and Figure 5) were similar to that of the Al samples. As the COF increased from zero, it then continues to oscillate. Nevertheless, it can be noted that the span of the variation in the HPT-processed Al MMCs was narrower than that of the PM Al MMCs. The PM Al 20%-Al_2_O_3_ and SiC COF values ranged from 0.25–1.35 and 0.25–1.25, respectively, which was larger than the 0.1–0.7 and 0.15–0.6 of the HPT-processed samples under a distance of 315 m. A similar observation was noted for the lower range of the COF for the HPT-processed Al MMCs compared to that for the PM COF under a distance of 1260 m (Figure 5).

**Figure 6:**
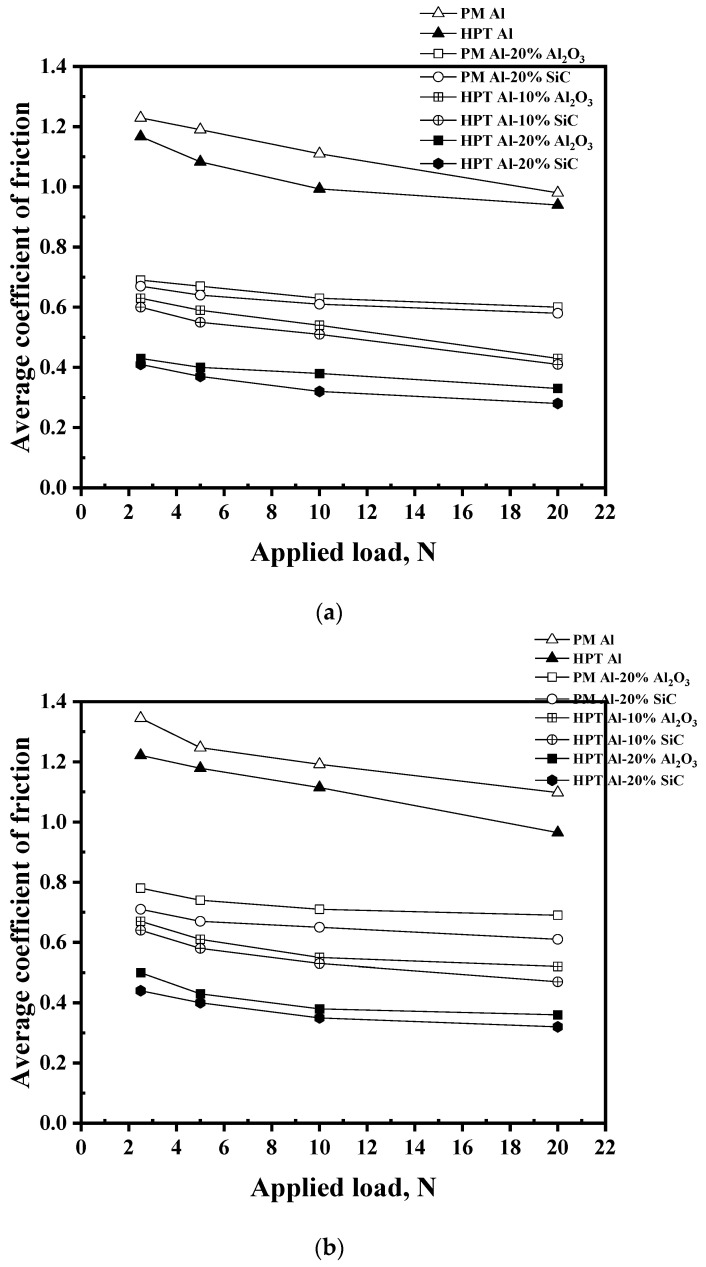

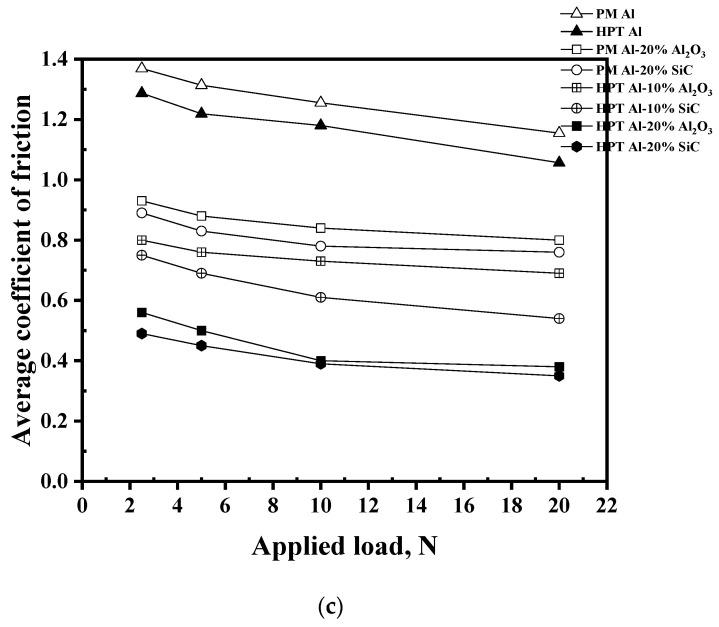
Average COF of the different samples under different applied loads and sliding distances of (**a**) 315, (**b**) 630, and (**c**) 1260 m.

According to the relationship with applied load, the average COF values decreased with the increase in the load under different distances (Figure 6). The decreasing COF values with the increase in load are congruent with that noted previously for severely processed MMCs and metals [34,37,38,40,41,42]. In contrary to the load, increasing the distance increases the COF [34,37,38,42]. In the formation of the Al MMCs, the increases in the reinforcement Vol% and the HPT processing decrease the COF. In the formation of the Al MMCs using PM and HPT processing, the COF values decreased by 43.9–47.2 and 66.1–70.2% (Figure 6), as noted previously [5,33]. Moreover, the COF of the HPT Al MMCs decreased by 31–37.3 and 23.3–45.2% after increasing the Al_2_O_3_ and SiC Vol% from 10 to 20%, respectively. Furthermore, the HPT processing of the Al-20% Al_2_O_3_ and SiC samples decreased the COF by 35.9–52.5 and 38–54% of that of the PM samples. The COF decrease after HPT processing is due to the microhardness, with a more noticeable effect relative to the reinforcement Vol% [26].

### 3.3. Worn Surface Morphology

**Figure 7:**
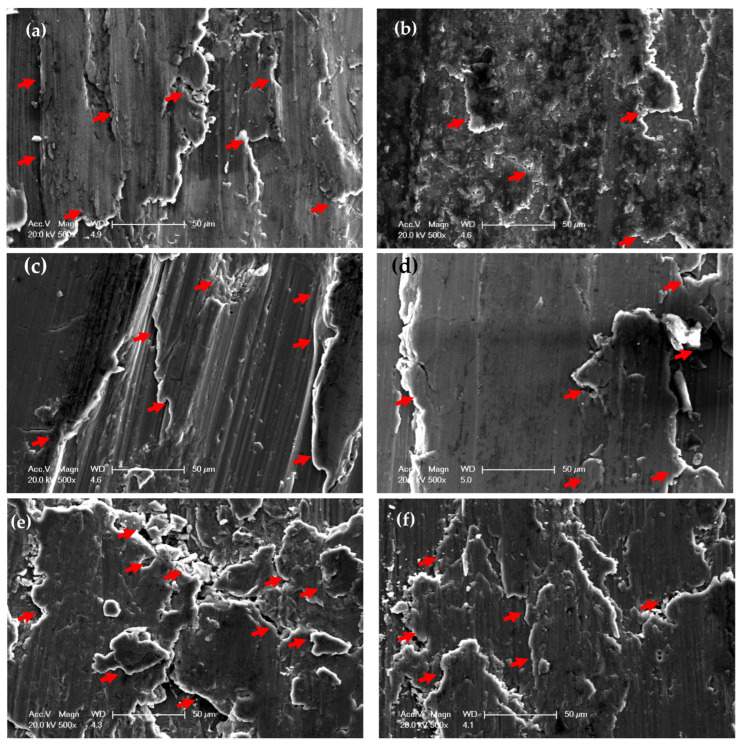
Worn surface morphology of (**a**,**c,e**) PM Al and (**b**,**d**,**f**) of HPT-processed Al, samples (**a**,**b**) under an applied load of 2.5 N and a sliding distance of 315 m, (**c**,**d**) under an applied load of 20 N and a sliding distance of 315 m, and (**e**,**f**) obtained under an applied load of 20 N and a sliding distance of 1260 m.

**Figure 8:**
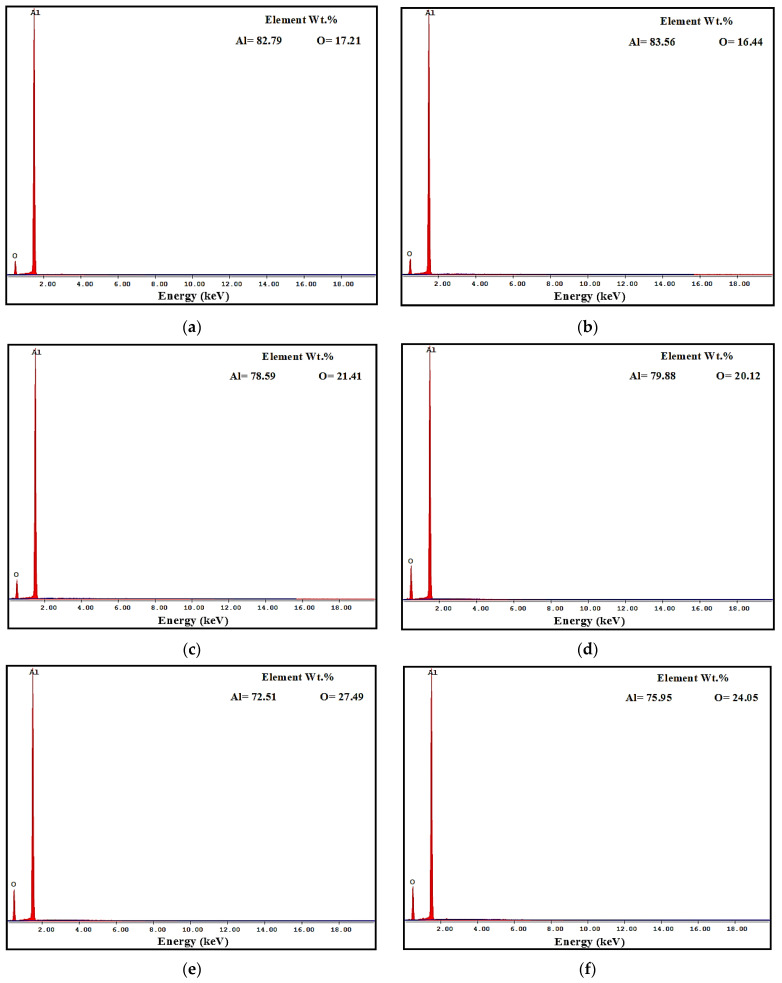
EDS analysis of worn surface morphology of (**a**,**c**,**e**) PM Al and (**b**,**d**,**f**) of HPT-processed Al samples (**a**,**b**) under an applied load of 2.5 N and a sliding distance of 315 m, (**c**,**d**) under an applied load of 20 N and a sliding distance of 315 m, and (**e**,**f**) obtained under an applied load of 20 N and a sliding distance of 1260 m.

Worn surface morphologies and analysis of the PM and HPT-processed Al samples are shown in Figure 7 and Figure 8. The wear mechanism of the Al samples was delamination and adhesion mechanisms, as shown by red arrows and as previously noted [31]. The degree of delamination of the different samples increased with both the load and distance. However, the delamination mechanism was more noticeable in the PM Al samples. The EDS analysis indicated the presence of oxygen in the Al samples (Figure 8). Therefore, oxidation can be considered a wear mechanism for the PM and HPT-processed Al samples. Interestingly, the oxygen content in the HPT-processed Al samples was lower than in the PM Al samples. The decrease in delamination, adhesion, and oxidation wear of the Al samples after the HPT processing can be explained by the increase in microhardness [26], as observed previously after the SPD of Al and Al alloys [34,35,37,38,42].

**Figure 9:**
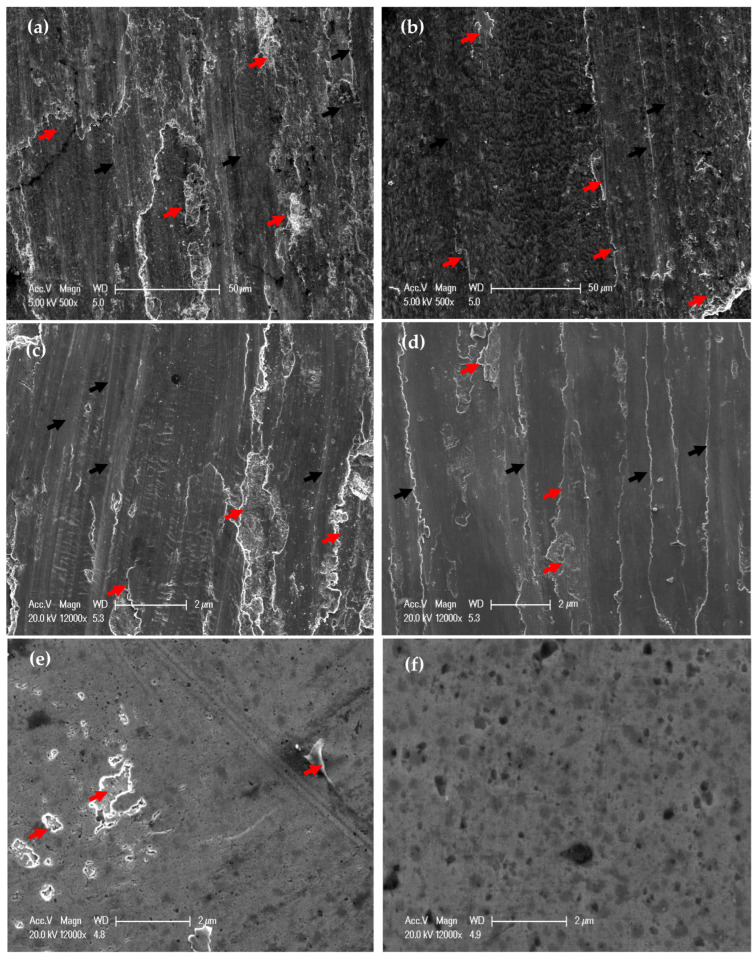
Worn surface morphology of (**a**) PM Al-20% Al_2_O_3_, (**b**) PM Al- 20% SiC, (**c**) HPT-processed Al-10% Al_2_O_3_, (**d**) HPT-processed Al-10% SiC, (**e**) HPT-processed Al-20% Al_2_O_3_, and (**f**) HPT-processed Al-20% SiC samples obtained under an applied load of 2.5 N and a sliding distance of 315 m.

**Figure 10:**
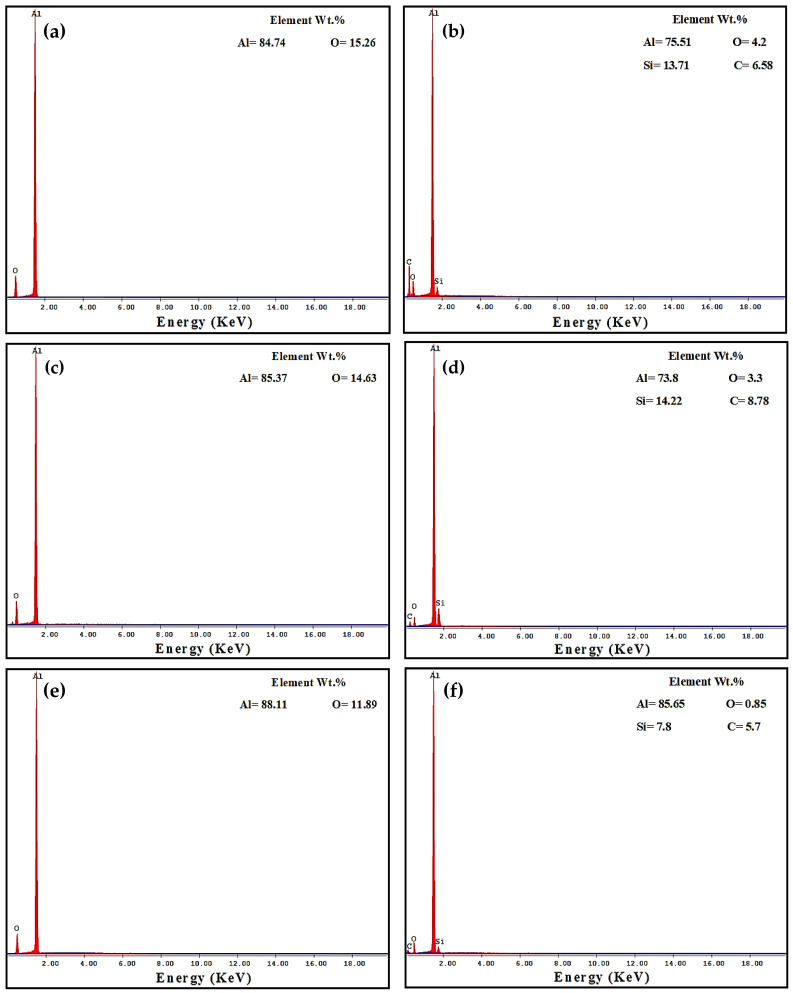
EDS analysis of the worn surface morphology of (**a**) PM Al-20% Al_2_O_3_, (**b**) PM Al-20% SiC, (**c**) HPT-processed Al-10% Al_2_O_3_, (**d**) HPT-processed Al-10% SiC, (**e**) HPT-processed Al-20% Al_2_O_3_, and (**f**) HPT-processed Al-20% SiC samples obtained under an applied load of 2.5 N and a sliding distance of 315 m.

**Figure 11:**
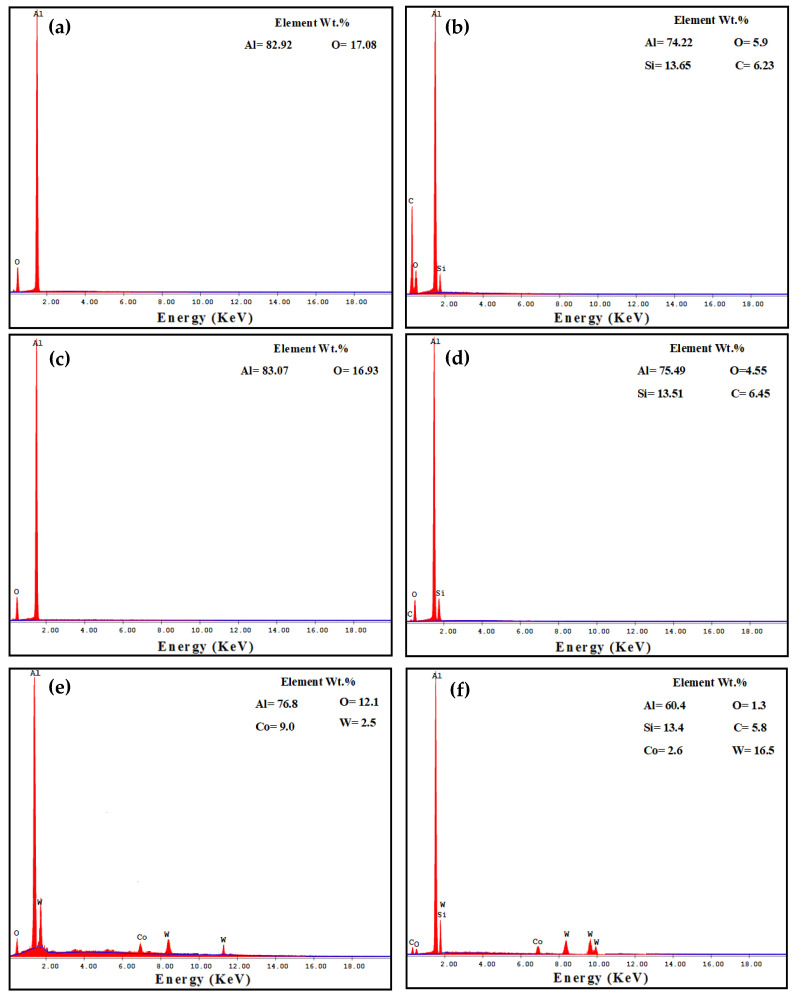
EDS analysis of the worn surface morphology of (**a**) PM Al-20% Al_2_O_3_, (**b**) PM Al-20% SiC, (**c**) HPT-processed Al-10% Al_2_O_3_, (**d**) HPT-processed Al-10% SiC, (**e**) HPT-processed Al-20% Al_2_O_3_, and (**f**) HPT-processed Al-20% SiC samples obtained under an applied load of 20 N and a sliding distance of 315 m.

**Figure 12:**
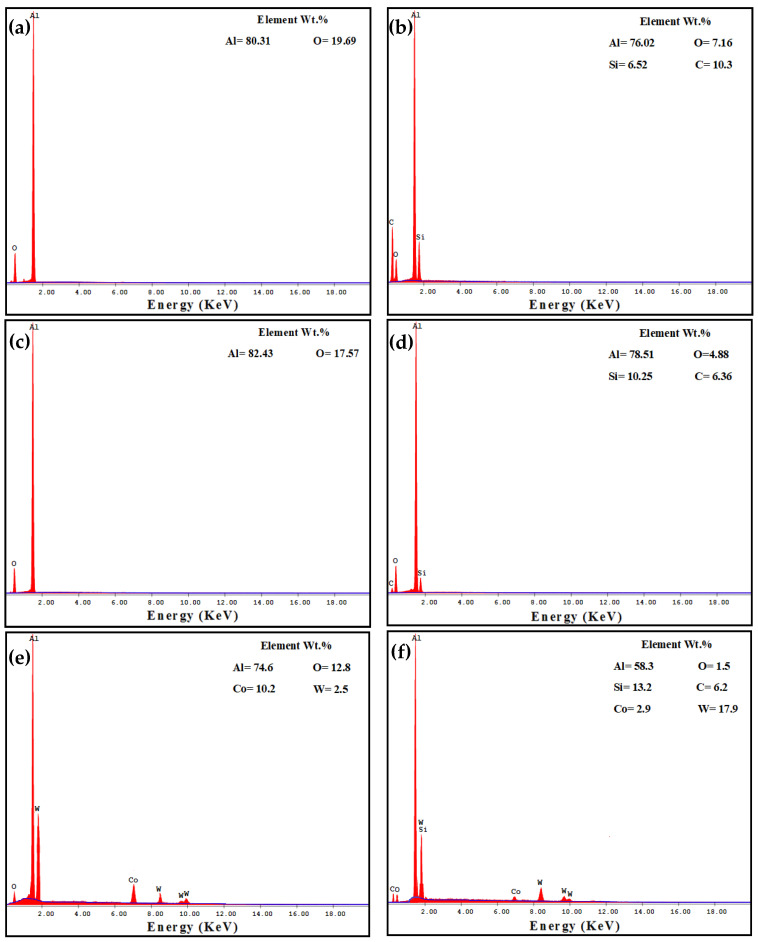
EDS analysis of the worn surface morphology of (**a**) PM Al-20% Al_2_O_3_, (**b**) PM Al-20% SiC, (**c**) HPT-processed Al-10% Al_2_O_3_, (**d**) HPT-processed Al-10% SiC, (**e**) HPT-processed Al-20% Al_2_O_3_, and (**f**) HPT-processed Al-20% SiC samples obtained under an applied load of 20 N and a sliding distance of 1260 m.

The PM Al MMCs wear mechanisms were adhesive, delamination (shown by red arrows), and shear streams along the sample (shown by black arrows) (Figure 9a,b). The delamination and adhesive degree of wear decreased after the HPT processing due to the increase in microhardness [26]. With further increases in the microhardness [26], the wear mechanism becomes abrasive and adhesive. The samples’ surfaces were almost free from delamination or taken off the reinforcement particles in the case of the HPT-processed Al-20% Alp2O3 and SiC samples (Figure 9e,f). The oxygen content also appeared in the Al MMC samples (Figure 10, Figure 11 and Figure 12), which confirms oxidation as a wear mechanism for Al MMCs, as previously noted [42]. Nevertheless, the oxygen content of the HPT-processed samples was smaller than that of the PM samples.

**Figure 13:**
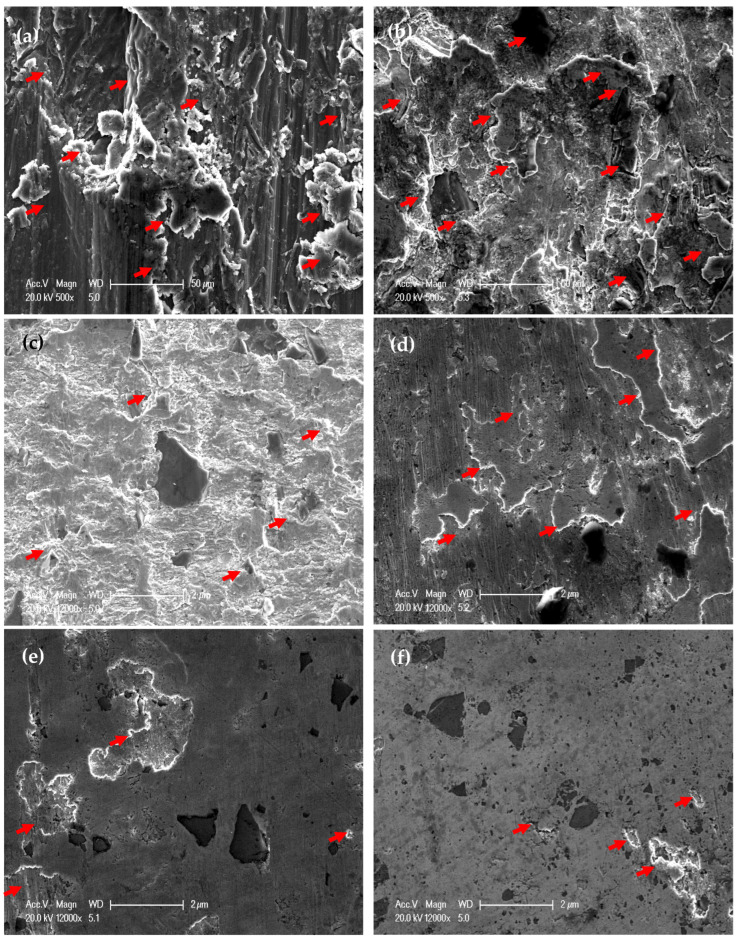
Worn surface morphology of (**a**) PM Al-20% Al_2_O_3_, (**b**) PM Al-20% SiC, (**c**) HPT-processed Al-10% Al_2_O_3_, (**d**) HPT-processed Al-10% SiC, (**e**) HPT-processed Al-20% Al_2_O_3_, and (**f**) HPT-processed Al-20% SiC samples obtained under an applied load of 20 N and a sliding distance of 315 m.

**Figure 14:**
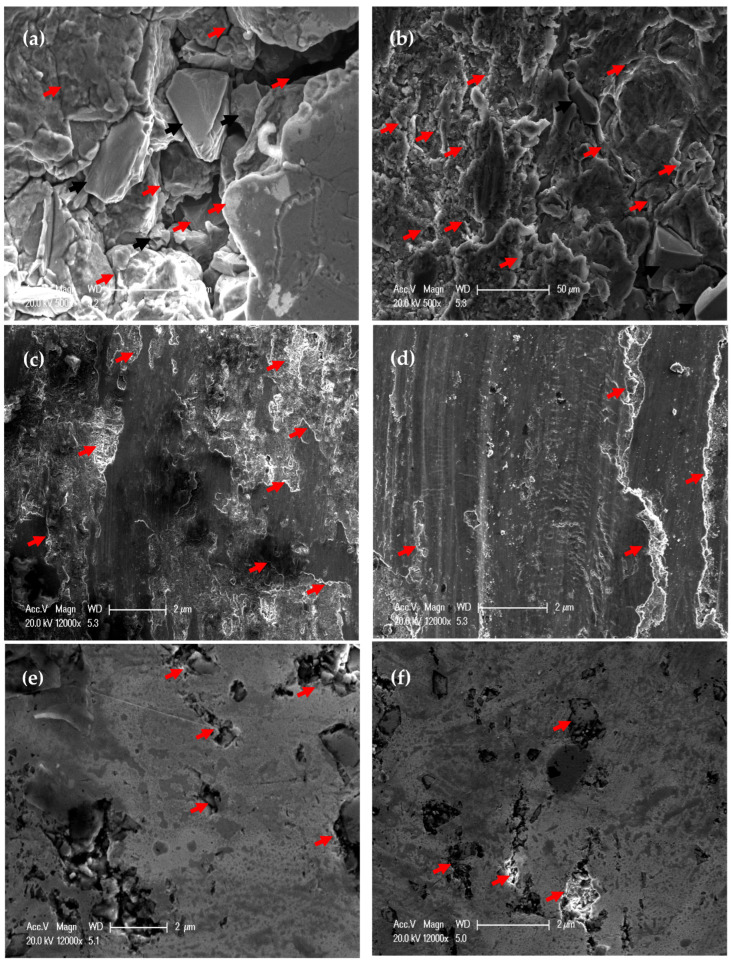
Worn surface morphology of (**a**) PM Al-20% Al_2_O_3_, (**b**) PM Al-20% SiC, (**c**) HPT-processed Al-10% Al_2_O_3_, (**d**) HPT-processed Al-10% SiC, (**e**) HPT-processed Al-20% Al_2_O_3_, and (**f**) HPT-processed Al-20% SiC samples obtained under an applied load of 20 N and a sliding distance of 1260 m.

Increasing the distance and load contributes to increasing both the wear rate and COF, as seen in (Figure 2 and Figure 6), which consequently increases the deterioration of the Al MMC samples surfaces (Figure 13 and Figure 14). However, the increase in the microhardness, remarkably after HPT processing [26], decreases the degree of surface damage. The increase in the load and distance, up to 1260 m and 20 N, destroy the surface of the PM Al MMCs, as indicated by the red arrows. (Figure 13 and Figure 14a,b). Although a small degree of damage was noted in the HPT-processed sample, free reinforcement particles were noted on the surface of the PM samples, as indicated by the black arrows (Figure 14a,b). The free reinforcement particles noted on the surface of the PM samples indicate the broken bond between them and the Al matrix.

On the other hand, the reinforcement particles appear to be still bonded to the Al matrix on the HPT-processed Al MMC sample’s surface. Thus, HPT-processed Al MMCs samples can be described as being the most wear-resistant. De-bonding of the reinforcement particles in the PM Al MMC samples contributes to a higher wear rate due to the occurrence of the three-body wear mechanism between the sample surface, WC ball, and reinforcement particles. This mechanism increases the wear rate and COF, which consequently deteriorates the PM samples surface relative to the HPT-processed surfaces, as previously noted [40,41]. 

The EDS analysis indicates the W transfer to the HPT-processed Al MMC sample surfaces (Figure 11 and Figure 12e,f). This observation confirms the high microhardness of the HPT-processed Al MMCs samples, which enables them to affect and erode the ball (as noted through WC ball morphology observations). Therefore, the particles were eroded from the W ball and transferred to the wear samples. Interestingly, the W content on the surfaces of the HPT-processed Al-20% Al_2_O_3_ and SiC samples increased with distance and load. 

A similar observation for the reduced wear rate of the Al, Al alloys, and Al MMCs after the SPD processing was noted through the worn surface observations, as also previously noted [29,31,32,33,34,35,37,38,42]. The SPD Al, Al alloys, and Al MMC wear surfaces were less affected by wear, with a lower degree of deterioration [33,34,35,37,38,42]. On the other hand, heavily destroyed sample surfaces were noted for samples processed by PM and casting and were also noted in previous work [29,31,32]. Therefore, SPD processing of the metals, metals alloys, and composites can effectively improve their wear characteristics.

### 3.4. Morphology of WC Ball Surface and Wear Debris

**Figure 15:**
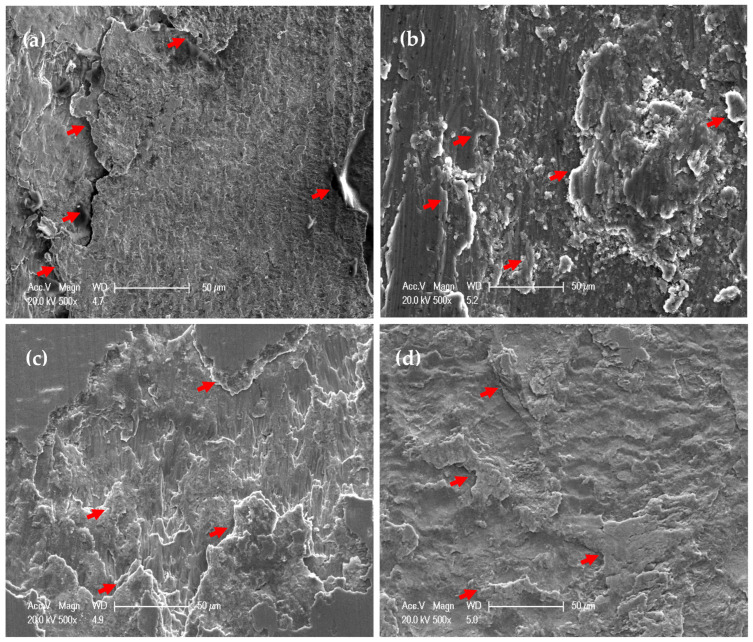
Worn surface morphology of the WC ball after sliding against (**a**) PM Al-20% Al_2_O_3_, (**b**) PM Al-20% SiC, (**c**) HPT-processed Al-20% Al_2_O_3_, and (**d**) HPT-processed Al-20% SiC samples under an applied load of 20 N and a sliding distance of 1260 m.

**Figure 16:**
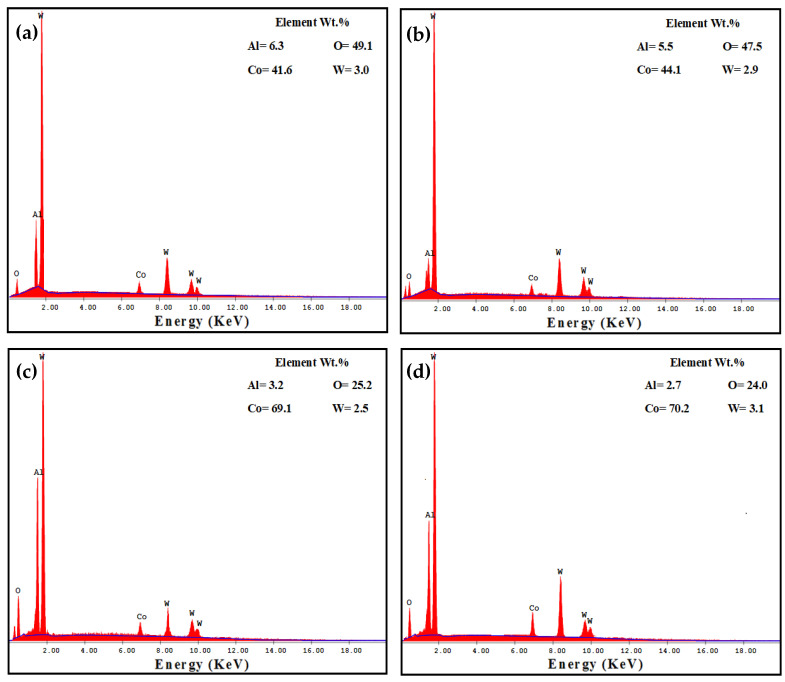
EDS analysis of the WC ball after the sliding against (**a**) PM Al-20% Al_2_O_3_, (**b**) PM Al-20% SiC, (**c**) HPT-processed Al-20% Al_2_O_3_ and (**d**) HPT-processed Al-20% SiC samples under an applied load of 20 N and a sliding distance of 1260 m.

The wear ball worn surface morphology and analysis after the test against the PM and HPT-processed Al MMCs under 1260 m and 20 N are shown in (Figure 15 and Figure 16). The WC ball surface can be characterized by the Al stick layer in the different cases, as noted through the analysis indicated by red arrows (Figure 16). However, the Al content was different from one case to another. The Al content was 6.3 and 5.5% in the PM Al-20% Al_2_O_3_ and SiC samples, respectively. On the other hand, the Al content was 3.2 and 2.7% after the wear test for the HPT-processed samples. Therefore, the PM samples were softer than the HPT-processed samples, which increased the Al layer stick on the ball. Moreover, the profound observations through SEM indicate that the Al layer was more obvious in the PM and approximately covered all of the ball surface (Figure 15a,b). 

The ball surface from the wear test with HPT-processed samples was covered in particular areas with stuck Al particles, as indicated by red arrows. However, other areas suffered from abrasive wear (Figure 15c,d). The presence of such eroded areas indicated the transfer of the W particles from the ball surface. Then, with further sliding, the W particles were attached to the sample surfaces, as confirmed by the EDS analysis (Figure 11 and Figure 12e,f). The obtained observations confirm the results of the higher microhardness [26] and superior wear resistance of the Al MMC samples processed using HPT, as previously noted [37,40,41,42].

**Figure 17:**
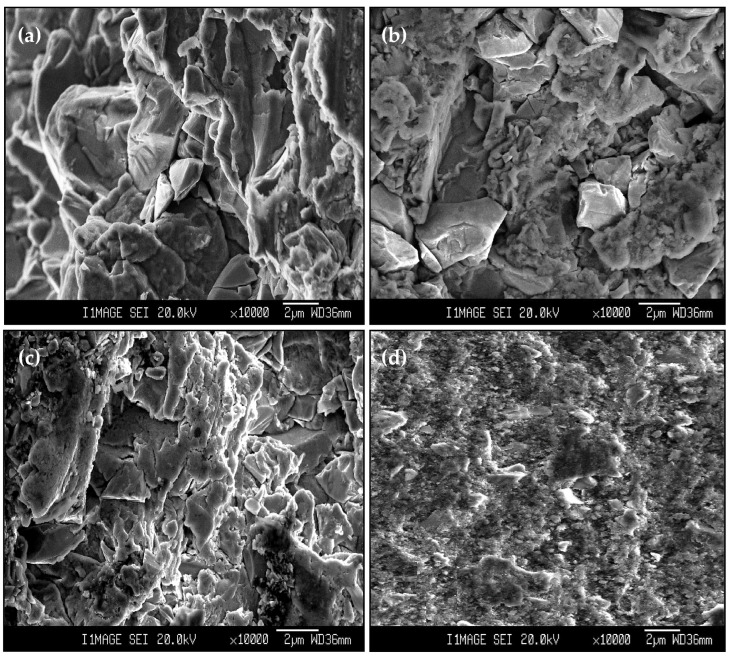
The wear debris of (**a**) PM Al-20% Al_2_O_3_, (**b**) PM Al-20% SiC, (**c**) HPT-processed Al-20% Al_2_O_3_, and (**d**) HPT-processed Al-20% SiC samples obtained under an applied load of 20 N and a sliding distance of 1260 m.

A large wear debris particle size was noted after the wear test on the PM Al MMCs (Figure 17a,b). The Al_2_O_3_ and SiC particles were noted clearly with their initial sizes [26]. The large Al particles confirm the higher removal amounts and wear rate in the PM Al MMCs. A noticeable decrease in the debris particle size was noted after the wear test of the HPT-processed Al MMCs (Figure 17c,d). The small wear debris from the HPT-processed Al MMC samples is due to the high microhardness of the HPT-processed samples [26], as previously noted after wear tests of HPT-processed Cu MMCs [40].

## 4. Conclusions

Through the present work, the following was concluded:

1. The formation of Al MMCs using PM and HPT processing decreases the wear scar widths of Al by 76–78 and 80–83% and its wear rate by 72–85.2 and 87.8–95.8%.
2. HPT processing of Al and Al MMCs significantly enhances their wear resistance. The HPT processing increased the Al and Al MMC samples’ wear resistance over those of the PM counterparts by 16–81%.
3. The HPT processing of Al and Al MMCs reduces their COF values and improves their frictional properties.
4. The Al and Al MMC processing methods affected the wear mechanism and worn surface morphologies. The worn surface microstructure observations and analyses of the samples and the WC balls proved the wear mechanism results.

## Data Availability

The data presented in this study are available on request from the corresponding author. The data are not publicly available due to the extremely large size.
